# Annotations of novel antennae-expressed genes in male *Glossina morsitans morsitans* tsetse flies

**DOI:** 10.1371/journal.pone.0273543

**Published:** 2022-08-29

**Authors:** Billiah K. Bwana, Paul O. Mireji, George F. Obiero, Consolata Gakii, Modesta O. Akoth, Julius N. Mugweru, Franklin N. Nyabuga, Benson M. Wachira, Rosemary Bateta, Margaret M. Ng’ang’a, Ahmed Hassanali

**Affiliations:** 1 Biotechnology Research Institute, Kenya Agricultural and Livestock Research Organization, Kikuyu, Kenya; 2 Department of Biological Sciences, University of Embu, Embu, Kenya; 3 Department of Biochemistry and Biotechnology, Technical University of Kenya, Nairobi, Kenya; 4 Department of Computing and Information Technology, University of Embu, Embu, Kenya; 5 Department of Chemistry, School of Pure and Applied Sciences, Kenyatta University, Nairobi, Kenya; Newcastle University, UNITED KINGDOM

## Abstract

Tsetse flies use antennal expressed genes to navigate their environment. While most canonical genes associated with chemoreception are annotated, potential gaps with important antennal genes are uncharacterized in *Glossina morsitans morsitans*. We generated antennae-specific transcriptomes from adult male *G*. *m*. *morsitans* flies fed/unfed on bloodmeal and/or exposed to an attractant (ε-nonalactone), a repellant (δ-nonalactone) or paraffin diluent. Using bioinformatics approach, we mapped raw reads onto *G*. *m*. *morsitans* gene-set from VectorBase and collected un-mapped reads (constituting the gaps in annotation). We *de novo* assembled these reads (un-mapped) into transcript and identified corresponding genes of the transcripts in *G*. *m*. *morsitans* gene-set and protein homologs in UniProt protein database to further annotate the gaps. We predicted potential protein-coding gene regions associated with these transcripts in *G*. *m*. *morsitans* genome, annotated/curated these genes and identified their putative annotated orthologs/homologs in *Drosophila melanogaster*, *Musca domestica* or *Anopheles gambiae* genomes. We finally evaluated differential expression of the novel genes in relation to odor exposures relative to no-odor control (unfed flies). About 45.21% of the sequenced reads had no corresponding transcripts within *G*. *m*. *morsitans* gene-set, corresponding to the gap in existing annotation of the tsetse fly genome. The total reads assembled into 72,428 unique transcripts, most (74.43%) of which had no corresponding genes in the UniProt database. We annotated/curated 592 genes from these transcripts, among which 202 were novel while 390 were improvements of existing genes in the *G*. *m*. *morsitans* genome. Among the novel genes, 94 had orthologs in *D*. *melanogaster*, *M*. *domestica* or *An*. *gambiae* while 88 had homologs in UniProt. These orthologs were putatively associated with oxidative regulation, protein synthesis, transcriptional and/or translational regulation, detoxification and metal ion binding, thus providing insight into their specific roles in antennal physiological processes in male *G*. *m*. *morsitans*. A novel gene (GMOY014237.R1396) was differentially expressed in response to the attractant. We thus established significant gaps in *G*. *m*. *morsitans* genome annotation and identified novel male antennae-expressed genes in the genome, among which > 53% (108) are potentially *G*. *m*. *morsitans* specific.

## Introduction

Management of Human African Trypanosomiasis (HAT) and Animal African Trypanosomiasis (AAT) initiative is a longstanding development preoccupation in sub-Saharan Africa, with control of different species of tsetse fly vectors considered a potentially effective means of disease control and suppression. All tsetse fly species are susceptible to different trypanosome infections and can transmit HAT and/or AAT to humans and livestock respectively. The critical determinant of differential transmission of trypanosomiasis among tsetse fly species is hinged on their host preferences mediated mainly by antennae resident olfactory processes in the different species. Tsetse flies typically navigate their environment by detecting and responding to volatile and non-volatile cues and display polymorphic responses between species, sexes, and allopatric populations [1–7]. Different tsetse flies transmit different trypanosome species depending on the hosts they prefer to feed on. For example, riverine tsetse species, such as *Glossina fuscipes fuscipes*, *Glossina palpalis* and *Glossina tachinoides*, prefer feeding on reptilian hosts, unlike the savannah species, such as *Glossina morsitans morsitans* and *Glossina pallidipes*, that feed largely on ungulates and other large mammals, a differential behavioural choice that is very likely associated with differences in their olfactory architectures. These olfactory attributes have been exploited in the characterization of tsetse fly species-specific attractant or repellent constituents and blends, which can be exploited for protection of humans and their livestock [8–10]. Further studies on structure-activity and blending of odor constituents have facilitated development of blends with enhanced attractance (‘pull’) [11] or repellence (‘push’) of savannah tsetse flies [12]. The findings of these studies have laid down useful groundwork for potential integration of repellent and attractant blends into ‘push-pull’ tsetse fly control strategies and provided opportunities for further studies on identification of more potent analogues and blends with enhanced ‘pull’ or ‘push’ effects.

Search for novel tsetse fly responsive compounds and blends (repellent or attractant) can be facilitated by characterizing molecular processes mediating species and/or sex specific differential responses. These can then be incorporated into existing optimized blends for possible enhancement of repellence or attraction to target tsetse fly species. Impetus to explore this novel approach is supported by availability of genomes of a suite of tsetse fly species (*G*. *m*. *morsitans*, *G*. *f*. *fuscipes*, *G*. *pallidipes*, *Glossina brevipalpis*, *Glossina austeni* and *Glossina palpalis gambiensis*) [13]. Initial efforts were focused on annotations of canonical chemosensory-active genes (coding odorant binding proteins, gustatory receptors, odorant receptors and ionotropic receptors) [14–17] and their expansions among these tsetse fly species [18]. Most of these chemosensory-active genes are expressed in the tsetse fly antennae, the principal olfactory system with diverse sensilla [19–20]. The antennae not only mediate chemoreception (detection of smell and taste), but also participate in mechanoreception, such as sensation of air-flow, vibration/sound, pressure changes, moisture and temperature [21–25], all of which mediate the overall responses of tsetse flies to external stimuli, including odors. Specifically, better understanding of genes putatively involved in these molecular events and/or their modulations, at molecular levels, could provide further insights on the processes directly or indirectly affecting responses of tsetse flies to odor cues, since chemoreception and mechanoreception integrate in their activity to regulate and co-ordinate tsetse fly responses to the stimuli.

Currently, six genomes of tsetse fly species have been annotated and published by the VectorBase community [13, 14]. Among these are active genes associated with chemoreception [14, 17]. These annotations, especially of *G*. *m*. *morsitans* genome, were however based in part on transcript evidence that were not antennae specific [13]. This suggests that the genes that are specifically expressed in the antennae, especially those with low expressions, were probably missed in the initial annotations because of their failure to meet the transcript expression threshold for inclusion in genome annotations and curations pipelines, such as MAKER [26] and Apollo software [27]. We hypothesized that antennae enriched transcriptome from adult male *G*. *m*. *morsitans* tsetse would be enriched with novel antennae expressed genes absent in the current annotations of *G*. *m*. *morsitans* genome in VectorBase [28]. Thus, in this study we sought to initially focus on male *G*. *m*. *morsitans* because males are available in larger proportions, compared to females that were limited by need for their deployment in the sustenance of our tsetse fly colony. Secondly, the males equally sufficed as females or mixed gender to establish proof-of-concept in the application RNA-Seq approach, and our associated data analyses pipeline in the identification and filling of gaps in genome annotations. Once established, the concept can be adopted to improve annotation of female specific antennae expressed genes.

We thus initiated this study specifically to (1) establish gaps in annotation of antennae expressed genes in male *G*. *m*. *morsitans*, 2) annotate and curate genes associated with these gaps and 3) establish transcriptional responses of the novel genes associated with the gaps to feeding, an attractant (ε-nonalactone) or a repellent (δ-nonalactone) [29] to the flies. We selected to use unfed (hungry) tsetse flies of varying phenotypic/physiological states associated with attraction to hosts or avoidance of non-hosts, typically mediated by antennal responses. Tsetse flies in these phenotypic/physiological states potentially provide transcriptomes enriched with transcribed genes in response to these stimuli (attractant, repellent, hunger/feeding), which in turn can be targeted for development of potential novel tsetse responsive molecules. Odors and hunger typically elicit stimuli-specific antennal transcriptional responses in insect vector, as evidenced by transcriptional antennal responses among members of the *Anopheles gambiae* species complex and *Anopheles sinensis* mosquitoes to blood feeding [30, 31], and similar responses to odor in *Drosophila melanogaster* [32] and *G*. *m*. *morsitans* [33]. Herein we report our findings.

## Materials and methods

### Study insects and reagents

We obtained antennae from male *G*. *m*. *morsitans* tsetse fly colony maintained at Yale University, New Haven, CT, USA, insectary. The flies originated from a small population originally collected from Zimbabwe. The flies were maintained at 24°C and 50–60% relative humidity (RH) and received defibrinated bovine blood (commercially supplied by Hemostat Laboratories, Dixon, CA, USA), via an artificial feeding system every 48h [34, 35]. Samples of the flies comprised of 1–3 days old teneral male flie*s* fed on defibrinated bovine blood- (the first blood meal post-eclosion), to putatively prime their chemosensory apparatus. We sourced for δ-nonalactone repellent (98–99% pure) as racemic mixture from Sigma-Aldrich (Taufkirchen, Germany) and synthesized racemic blend of ε-nonalactone attractant (not commercially available) in the laboratory as previously described [6]. We confirmed the structure of ε-nonalactone by High Resolution Mass Spectrometry (HR-MS), carbon 13 Nuclear Magnetic resonance (^13^C NMR), Hydrogen Nuclear Magnetic Resonance (^1^H-NMR), and Fourier Transform Infrared (FTIR) spectrophotometry as detailed and reported in Wachira et al. [29]. We tested all odorants at 10^−3^ dilutions in paraffin oil (1% vol/vol), as previously used in the assessment of laboratory responses of *G*. *m*. *morsitans* to odors [33].

### Collection of male *G*. *m*. *morsitans* tsetse fly antennae samples

We collected the blood fed teneral male *G*. *m*. *morsitans* (1–3 days old) and starved some for 72hrs to ‘induce’ hunger and potentially prime them into ‘host seeking’ physiological state. We kept the other normally fed tsetse to assess the effect of feeding on antennal transcriptional changes. We hypothesized that the unfed flies would be conditioned to distinguish host (attractant) or non-host (repellent) odor compounds compared to paraffin oil as control. Briefly, we separately placed three independent replicates each consisting of 50 flies in 1L transparent glass jars, with 100 μl of the paraffin oil diluted attractant or repellent via Whatman filter paper (1 cm in diameter) [33]. We similarly exposed the unfed tsetse fly control groups to the paraffin oil diluent. In all cases, we enclosed the glass jar for five hours for optimal exposures of the flies (unfed, attractant or repellent exposed treatments) under insectary conditions. We assumed that the head space concentration of the attractant or repellent corresponded to 10^−3^ dilution in paraffin oil within each jar. We did not assess empirical prevalence/concentration of the odors in headspace. We similarly handled the fed flies, but in two independent replicates due to limitations in biological samples/materials. It has previously been demonstrated that five hours exposure is optimal for desensitization of receptors and subsequent transcriptional change in response to odors in *Dm* [32]. Since *G*. *m*. *morsitans* show marked diel changes in their biting activity in the field, with their peak activity in the mornings and afternoons [20], we performed the exposures from 7:00 am to 12:00 pm to encompass the morning sessions of their peak activities and extracted a pair of antennae from each fly and in each treatment (attractant/repellent exposed, fed or unfed (control)) as described by Kabaka et al. [18]. Briefly, we snap-froze the flies at the end of the exposure duration by placing the jars containing the flies in -80°C freezers. In each treatment, we carefully hand-dissected, pooled pairs of antennae from the head of each fly and placed them in 1.5 ml microfuge tubes (separated by replicate) kept in ultra-cold liquid nitrogen by methods of Menuz *et al*., [36]. We then isolated RNA by mechanically crushing the antennae with disposable RNAseq-free plastic pestles in TRIzol reagent (Invitrogen, Carlsbad, USA) following the manufacturer’s protocol. We removed traces of potential carry over DNA (that could potentially confound our RNA-Seq analysis) by digesting possible contaminating genomic DNAs (gDNA) in the total RNA using TURBO DNase (Ambion life technologies, TX, USA) following manufacturer’s instructions. We confirmed removal of the gDNA from total RNA by qualitative assessment of PCR amplicons from final RNA samples using tsetse specific *beta*-*tubulin* gene primers as documented in Bateta *et al*. [37]. We verified quality and integrity of RNA samples using Agilent Bioanalyzer 2100 (Agilent, Palo Alto, CA, USA) following manufacturer’s instructions. cDNA was then generated from the RNA using *Illumina* TruSeq RNA *Sample Preparation Kit (*Illumina, Hayward, CA, USA) and the cDNA (101 bp paired end read) sequenced on Illumina HiSeq 2500 at Yale University Center of Genome Analysis (YCGA), New Haven, CT, USA. We selected paired rather than unpaired-end read sequencing platform to facilitate subsequent more accurate alignment of the reads on our reference transcripts [38]. We thus sequenced eleven libraries consisting of three replicates each of antennae from attractant, repellent or control exposed flies, and two from antennae of fed flies. We deposited all these raw read sequences at the Sequence Read Archive (SRA) under study accession number PRJNA344035.

### Assessment of gaps in annotation of antennae expressed genes in male *G*. *m*. *morsitans* tsetse flies

We established quality of the reads in each individual transcriptome library using FastQC version 11.0 (Babraham Bioinformatics) software package (http://www.bioinformatics.babraham.ac.uk/projects/fastqc/). We then used the FastQC results to clean (trim) and remove low quality reads from respective transcriptomes using Trimmomatic software version 3.8 [39] that implemented 1) -phred33 scale of quality scores commensurate with our RNA-Seq data quality and format and 2) settings that permitted sequential cleaning of leading or trailing three nucleotides within 4:15 sliding window with at least 36 nucleotides. This cleaning process generated 1) paired reads of forward and their counterpart reverse reads surviving, 2) unpaired (orphaned) reads where the forward or reverse reads did not survive or 3) none, where neither forward nor reverse reads survived the cleaning process. We concatenated the forward or reverse components of the clean paired reads category from different treatments and replicates and mapped them (reads) onto *G*. *m*. *morsitans* transcripts gene-set version 1.9 or genome version 1.0 from Vectorbase [28] using Bowtie 2 ultrafast short sequence reads aligning software version 2.3.5 [40] with settings that also isolated unmapped reads from each mapping procedure. We hypothesized that differences in mapping statistics between the transcript and the genome would lend insight into the gap in annotation of the genome, with the genome mapping statistics providing additional insight on possible contamination of the original RNA (evidenced by poor mapping statistics) as an additional quality control procedure. We collected the unmapped paired reads from the transcript mapping procedure (associated with the potential gap in annotation), *de novo* assembled them (unmapped reads) into transcripts and assessed quality of the assembled transcripts using the short reads Trinity *de novo* assembly software 2.10.0 [41].

We mapped back our unmapped reads onto our *de novo* assembled transcripts using Bowtie 2 ultrafast short sequence reads aligning software version 2.3.5 [40] to establish the proportion of reads that were incorporated/employed in the *de novo* assembly, as a measure of efficiency of the assembly process. We isolated the longest transcripts with open reading frames (most representative of the respective genes) that could putatively yield peptides with at least 100 amino acids long using TransDecoder software [42]. We queried these transcripts for their putative functions/homologs in protein database UniProt release-2020-04 [43] or corresponding transcript in *G*. *m*. *morsitans* transcripts gene-set version 1.9 from VectorBase [28] using Basic Alignment Search Tool (BLAST) analysis for protein (tBlastx) or nucleotide (BLASTn) sequences [44] respectively. We considered our *de novo* transcript 1) a homolog of a Uniport database gene if it had an e-value < 0.001, a query coverage and length at least 95% and 100 amino acids respectively, and 2) corresponding transcript of *G*. *m*. *morsitans* transcript if it had an e-value < 0.001, query coverage and identity of at least 95% and length of 300 nucleotides. We considered transcripts with neither homologs nor corresponding transcripts in either database as novel transcripts. Those transcripts lent further insight into the gap in genome annotation. We separately used the longest transcripts with open reading frames to predict novel protein-coding genes in the *G*. *m*. *morsitans* genome version 1.0 from Vectorbase [28] using MAKER computational pipeline [26]. This pipeline employed *ab initio* gene predictions, transcript evidence and homologous protein evidence [26]. Our transcripts and Uniprot/Swiss-Prot protein database [43] publicly available at https://www.uniprot.org/ (accessed on 10 June 2020) served as transcript and protein evidence respectively within the pipeline. We finally assessed for proportion of our longest transcripts with open reading frames that MAKER used in the prediction of protein-coding genes by searching for the corresponding genes for our transcripts using BLASTn [44]. We considered *de novo* transcript correspondent with the predicted genes if the e-value was < 0.001, and the query coverage and identity were at least 95% each.

### Annotation of the novel expressed genes in male *G*. *m*. *morsitans* tsetse fly

We manually curated the final gene models generated by the MAKER software by inspecting and refining the precise gene structure and putative function in graphical browser-based curation Apollo software [27] platform in community VectorBase [28]. Our major steps in the manual curation included 1) investigating exon/intron structure integrity and setting start and/or stop coordinates based on our concatenated RNA-seq evidence track as well as existing tracks in VectorBase, 2) verifying consistency and accuracy of the curated gene models by querying them against known homologs in *D*. *melanogaster* within VectorBase [28] and 3), internal validation and provision of stable sequence identities/adopted by VectorBase [28]. We then assessed the improvement of our coverage in annotation of the genome by mapping the (original) concatenated reads onto combined transcript dataset consisting of *G*. *m*. *morsitans* gene-set (Var 1.9) [28] and the new annotated gene transcripts, using Bowtie 2 software version 2.3.5 [40]. We assessed the proportion of our *de novo* assembled transcripts utilized/accepted by MAKER computational pipeline in processing of the gene predictions by performing a nucleotide search using Basic Alignment Search Tool (BLASTn) [44] of the *de novo* assembled transcripts as query against the newly curated genes as subject. We isolated novel genes (predicted protein-coding genes) from among our newly annotated genes by performing a nucleotide search using BLASTn [44] of the annotated genes against *G*. *m*. *morsitans* gene-set (Var 1.9). We then identified putative functions of these novel genes (without corresponding gene in the *G*. *m*. *morsitans* gene-set (Var 1.9) by identifying their 1) homologs in Uniport/Swiss-prot protein database [43] using BLASTp [44] search against uniport protein database [43] (accessed on 10 June 2020), accepting hits with e-value < 0.001 as significantly homologous, 2) protein domains and GO terms associated with the predicted protein-coding genes using standalone InterproScan software version 5.52–86.0 [45] and 3) orthologs in *M*. *domestica* or *An*. *gambiae* mosquito genomes obtained from VectorBase release 53 [28] or *D*. *melanogaster* genome from FlyBase [46] using default settings in OrthoFinder software version 2.5.4 [47].

### Assessment of differential responses of the novel expressed genes to *G*. *m*. *morsitans* tsetse fly attractant or repellent odor cue

**Fig 1:**
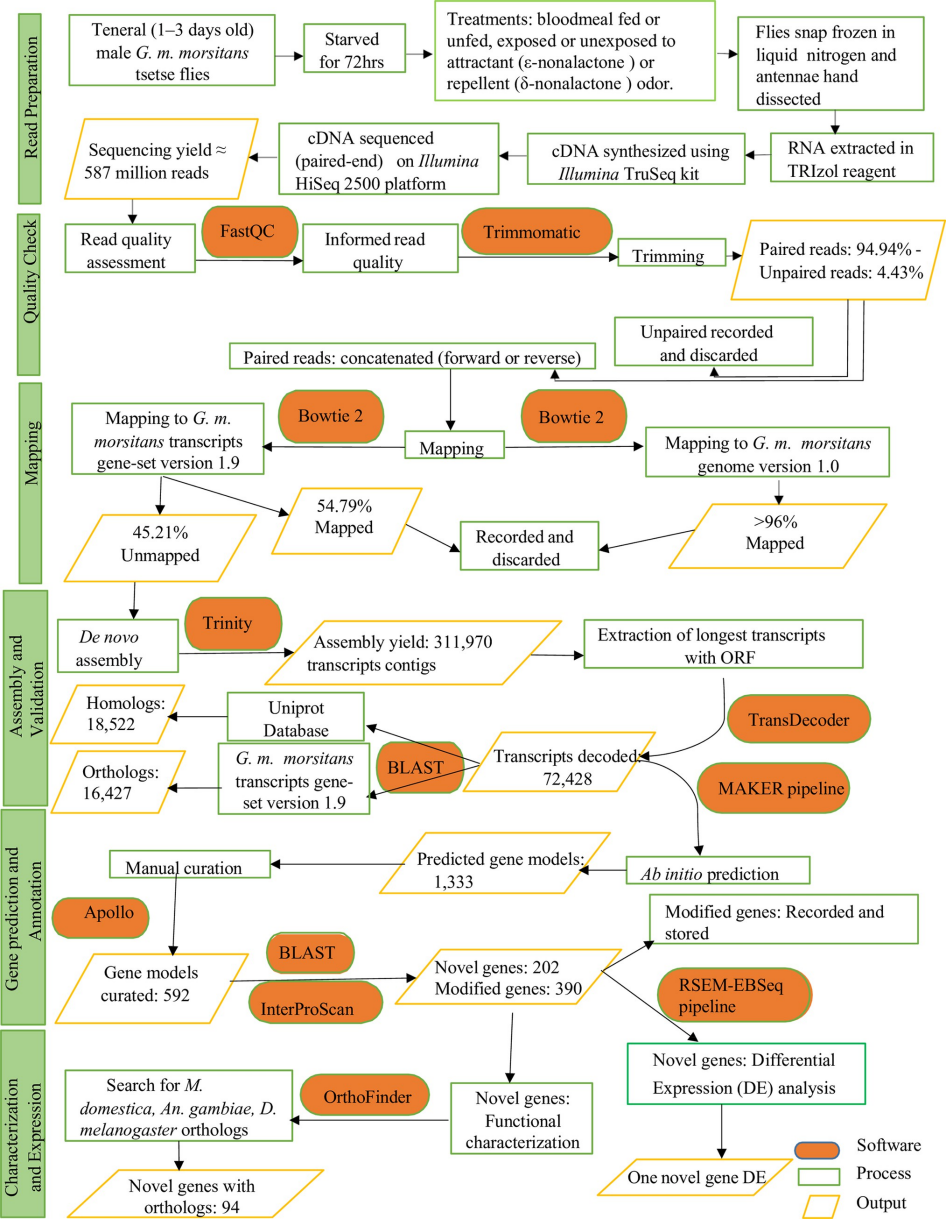
Schematic flowchart diagram of processing of samples and bioinformatics analysis of RNA-Seq transcriptome data from male *G*. *m*. *morsitans* tsetse fly antennae.

We assessed any differential expressions of the novel genes in response to the attractant (ε-nonalactone) or the repellent (δ-nonalactone) structural analogues, and compared these with the effect of feeding, using RSEM (RNA-Seq by Expectation Maximization) EBSeq pipeline [48]. Responses to the *G*. *m*. *morsitans* transcripts gene-set version 1.9 from VectorBase [28] to these odor molecules (ε-nonalactone/δ-nonalactone) have been previously evaluated and reported elsewhere [49]. In the present study, we built RSEM transcript references for our novel gene transcripts and separately mapped our clean paired reads and replicates from each of our treatment libraries (fed, unfed, exposed to the repellent or the attractant) individually onto our novel transcripts using Bowtie 2 version 2.3.5 [40]. We extracted the estimated read counts for respective transcripts or their isoforms from each library and replicate, and subsequently generated a count matrix from comparisons of the reads from attractant, repellent or fed treatment libraries to the unfed library (control) using RSEM–EBSeq pipeline [48]. This analysis thus generated a list of relative expression levels of each transcripts/isoform in the treatments relative to the control. We considered the transcripts/isoform significantly different if there was a post-fold change of at least 2X and false discovery rate (FDR) corrected p < 0.05 between other treatments/libraries relative to the unfed control. We established the GO term associated with differentially expressed gene(s) from VectorBase [28]. We have summarized how we generated and processed our data in a flowchart presentation in Fig 1.

## Results

### New antennae-specific transcripts establish annotation gaps in the *G*. *m*. *morsitans* tsetse fly genome

**Fig 2:**
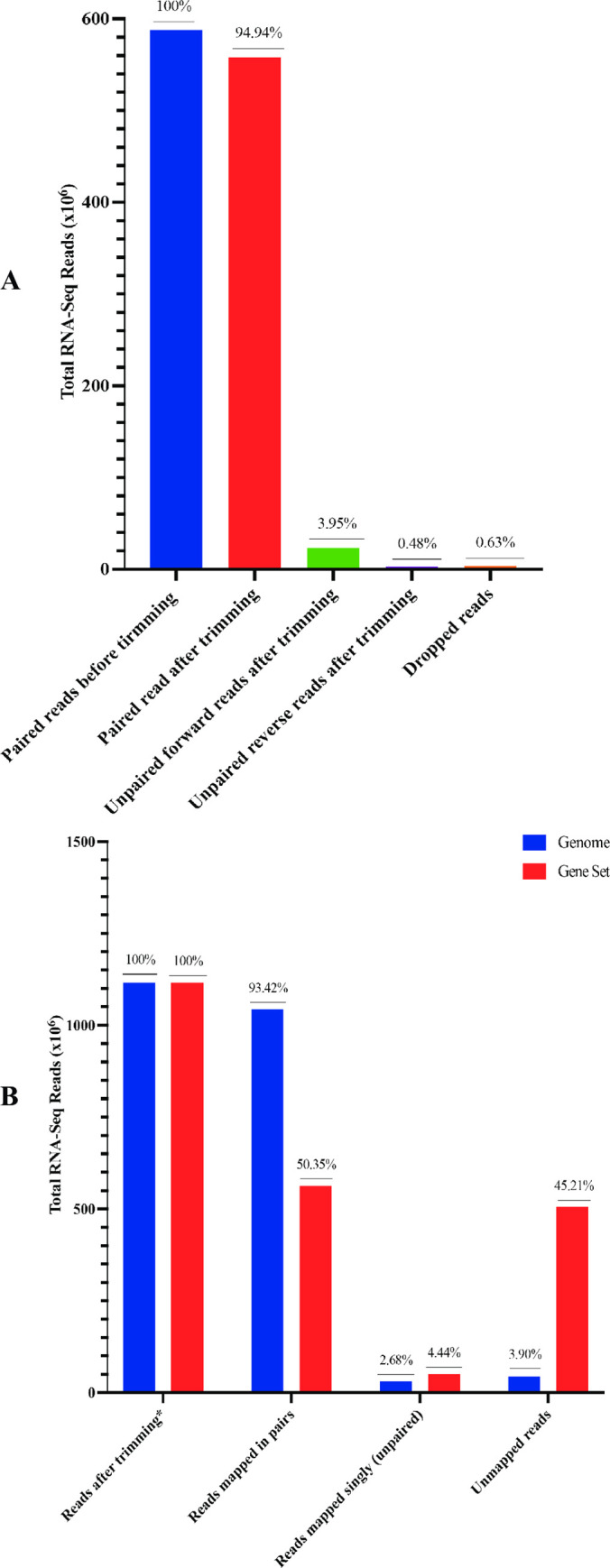
Quality and mapping statistics of concatenated RNA-Seq library from male *G*. *m*. *morsitans* tsetse fly antennae. Panel A. Quality assessment statistics of the libraries. We extracted total RNA from blood fed/unfed teneral male *G*. *m*. *morsitans* exposed/unexposed to attractant (ε-nonalactone) or repellent (δ-nonalactone) in two/three independent replicates. We sequenced the RNA on Illumina HiSeq 2500, concatenated the resultant reads into a single library and established quality of the library using FastQC v11.0 software package. We used the FastQC results to clean (trim) and remove low quality reads from respective transcriptomes using Trimmomatic software version 3.8 [39]. Panel B. Mapping statistics of the libraries. We mapped clean paired (concatenated) reads onto *G*. *m*. *morsitans* transcripts gene-set version 1.9 or genome version 1.0 from Vectorbase [28] using Bowtie 2 ultrafast short sequence reads aligning software version 2.3.5 [40] with settings that also isolated unmapped reads.

We obtained 587 million reads from sequencing all the total RNA libraries from the antennae of adult male *G*. *m*. *morsitans*. More than 99% of these libraries passed the quality control test as clean paired (94.94%) or unpaired (4.43%) reads (Fig 2). More than 96% of the clean paired reads mapped onto the genome, of which 93.42% mapped as pairs and 2.68% mapped singly (unpaired). However, only 54.79% of the clean paired reads mapped onto the *G*. *m*. *morsitans* gene set sequences, of which 50.35% mapped as pairs and 4.44% singly (unpaired) (Fig 2). Of the clean paired reads (described here as divergent), 45.21% mapped onto the genome but not onto the *G*. *m*. *morsitans* gene set. This was indicative of their *G*. *m*. *morsitans* origin (not contaminants or of parasitic nature), while potentially revealing the gap in the annotations of active genes in the genome. The less than 4% of the clean paired reads that did not map onto the genome potentially represent gaps in assembly of the genome and/or presence of symbionts or pathogens in the fly population.

### Divergent (unmapped) reads are associated with antennae-expressed novel genes in the male *G*. *m*. *morsitans* tsetse fly

**Fig 3:**
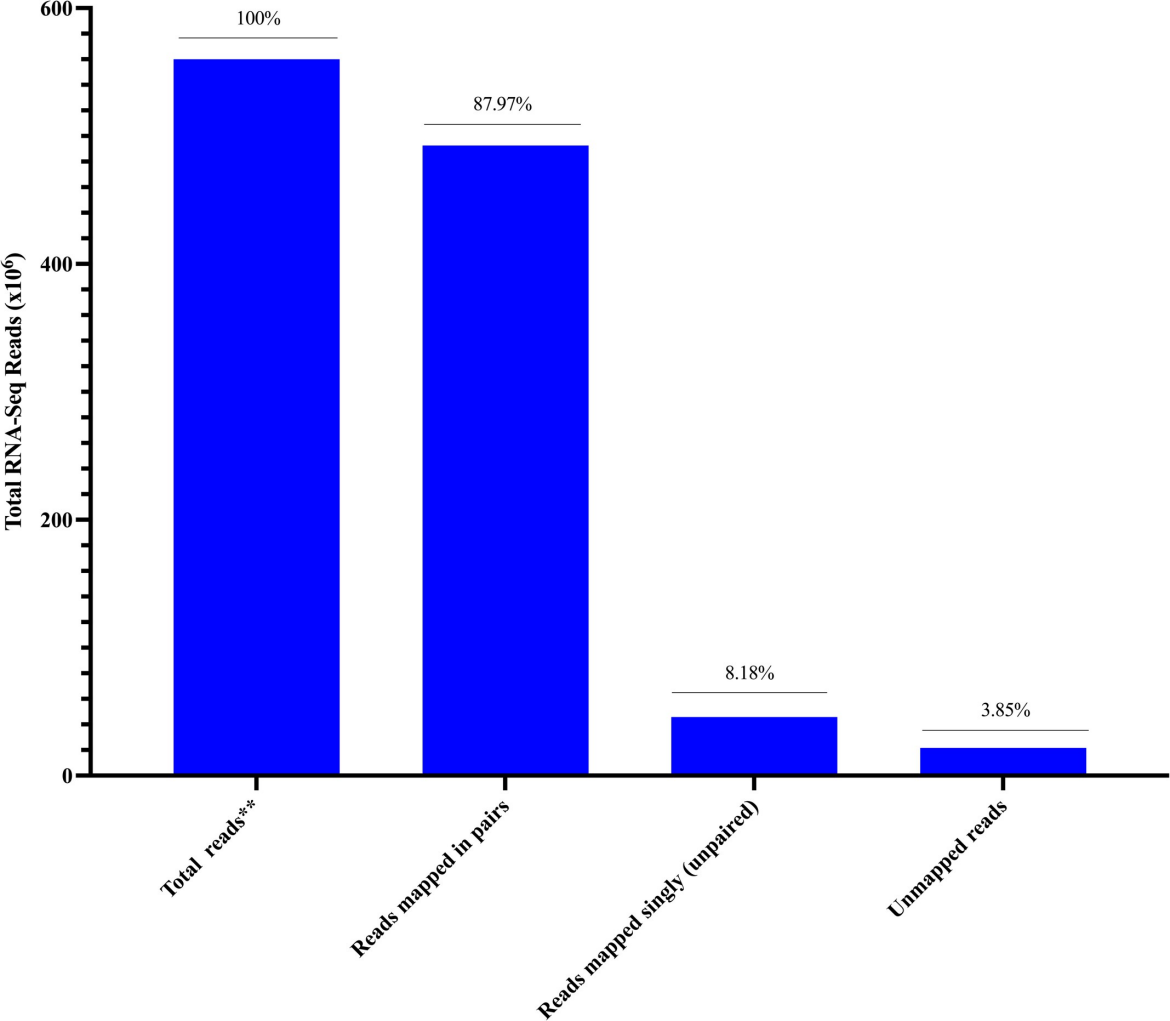
Quality assessment of read representation in *de novo* assembled antennal transcripts from male *G*. *m*. *morsitans* RNA-Seq antennal libraries. We *de novo* assembled the unmapped reads into transcripts and assessed quality of the assembled transcripts using short reads Trinity *de novo* assembly software 2.10.0 [41] that mapped the reads onto their respective assembled transcripts to account for individual reads (incorporation).

Our *de novo* assembly of the unmapped paired reads yielded to 311,970 transcript contigs, grouped into 213,184 putative genes. The assembly had a GC quality content of 34.18% and contig N50 quality statistic of 1,445 for all genes, and 903 (N50) for the longest isoforms. The median contig base length was 478 and 367 bases, with average base length of 857.16 and 626.51 bases for genes or longest isoforms respectively (Table 1). Mapping back our reads onto our *de novo* assembled transcripts revealed that at least 96.15% of our unmapped reads were used in construction of *de novo* transcripts, among which 87.97% of the reads mapped in pairs and 8.18% of the reads mapped singly (unpaired) (Fig 3). We isolated 72,428 non-redundant longest transcripts with open reading frames, and with an open reading frame at least 300 nucleotides (100 amino acids) long. Our search for the homologs of these transcripts in Uniprot database genes [43] revealed that only 25.57% (18,522) of these transcripts had corresponding homologs comprising of 5,252 unique (non-redundant) Uniprot database genes [43] (S1 Table). Similarly, only 22.68% (16,427) of the transcripts had corresponding transcripts in *G*. *m*. *morsitans* transcripts gene-set version 1.9 [28] comprising of 4,887 unique (non-redundant) genes, among which 48.58% (2,374) were annotated (S2 Table). Overall, these searches revealed that some of the *de novo* assembled transcripts associated with the unmapped reads (gaps in genome annotation) were potentially linked to functionally annotated genes, while others were novel without corresponding transcripts/genes in the databases. The functionally annotated genes homologous to assembled transcripts in both databases were putatively associated with chemoreception, regulation of gene expression and translation, detoxification of xenobiotics and responses to stress among other attributes (S1 and S2 Tables).

**Table 1:** Trinity transcriptome assembly quality assessment.

	Statistics	
Attribute	All Transcripts	Longest Isoform*
Genes	213184	-
Transcripts	311970	-
GC %	34.18	-
Contig N50	1445	903
Median contig length	478	367
Average contig length	857.16	626.51

**Fig 4:**
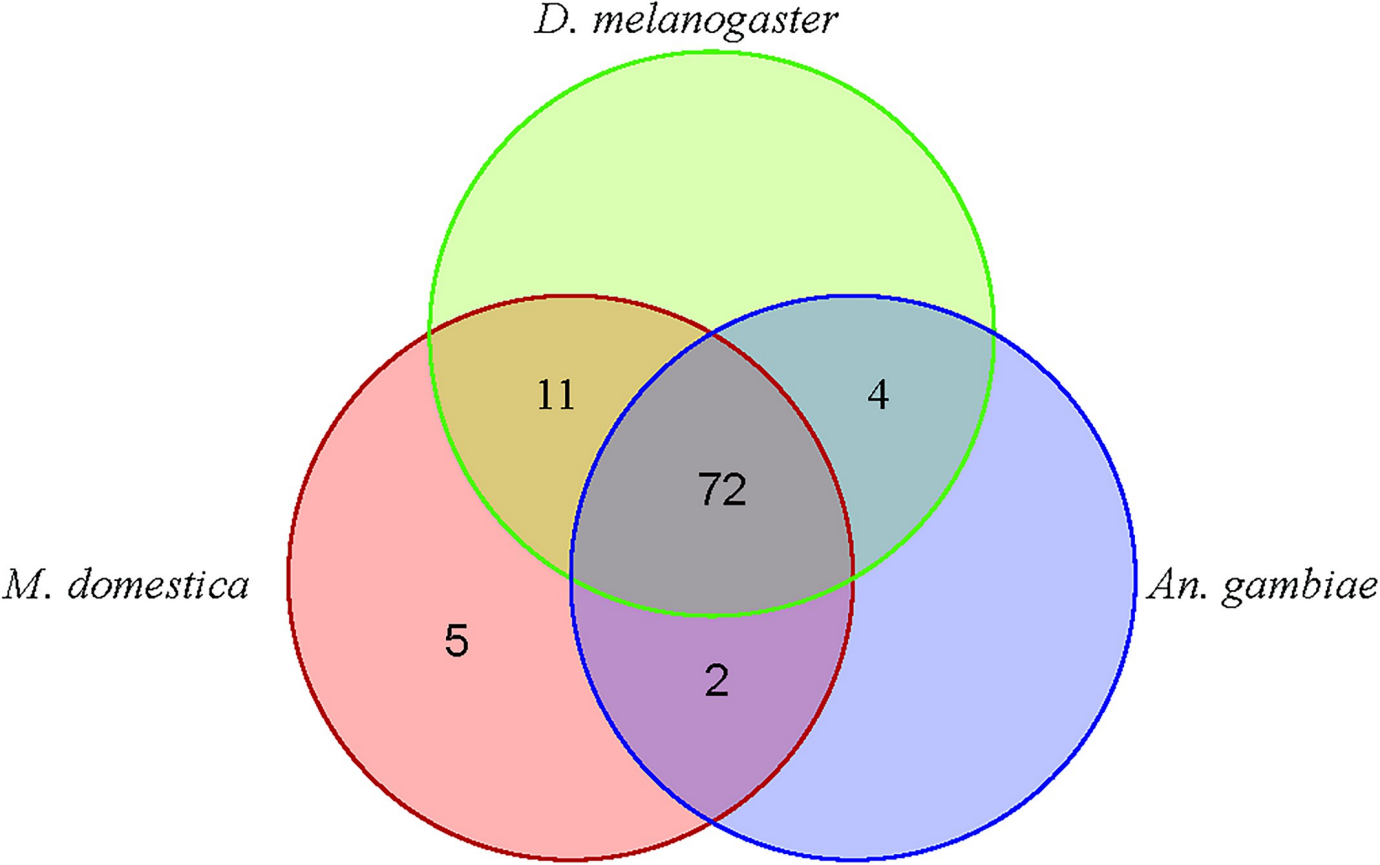
Orthologs of novel annotated male *G*. *m*. *morsitans* antennae genes in *D*. *melanogaster*, *M*. *domestica* and *An*. *gambiae genomes*.

Our prediction of protein-coding genes associated with the *de novo* assembled transcript in *G*. *m*. *morsitans* genome in Vectorbase [28] identified 1,333 gene models potentially associated with the divergent/unmapped reads. Assessment of the proportion of our *de novo* assembled transcripts used in the prediction of protein-coding genes of the transcripts revealed that 68.86% (214,835) of these were deployed in the predictions, and about 30% were not, likely due to insufficient evidence. Our interrogation of these predicted genes in a VectorBase community platform [28] established 73.74% (983) of the predicted genes as valid structural and putative functional genes, among which 592 models showed sufficient supportive evidence and were curated in the database. Among these, 202 models comprised of novel genes with the rest (390) constituting isoforms/variants of existing genes in Vectorbase [28]. Our search for the orthologs of the novel genes in *M*. *domestica*, *D*. *melanogaster* or *An*. *gambiae* genome revealed that 46.53% (94) of the novel genes had orthologs in at least one of the target genomes (S3 Table), with some orthologs shared among the genomes (Fig 4). The rest without orthologs were potentially novel in relation to the three genomes. These orthologs are putatively associated with cellular processes such as oxidative phosphorylation, protein synthesis, transcription and translation regulation, detoxification, lipid and carbohydrates metabolism, embryogenesis, male courtship regulation, neural cell adhesion, protein folding, metal ion binding, G protein coupled receptor signaling, learning and memory development, immunity induction, protein degradation and protein hydrolysis, among others (S3 Table). Similarly, analysis of the novel genes within the Uniprot database [43] established that 43.56% (88) had potential homologs in the database (S4 Table). These homologs were putatively associated with amino acid biosynthesis, transcriptional regulation, memory formation, oxidative regulation, lipid metabolism, ribonucleic acids processing, stress response, signal transduction, metal ion binding, embryo development and protein folding, modification, hydrolysis and degradation functions, among others. Both analyses thus revealed that most of the novel genes (> 53%) were potentially *G*. *m*. *morsitans* specific and appeared to be in sync with our initial analyses with the *de novo* assembled transcript above. Our search for protein domains and GO terms associated with these novel genes category revealed protein kinase domain, autophagy-related protein C terminal, nitrogen permease regulator 2, beta-acetyl like hexosaminidase, RNA recognition motif, ubiquitin family, ribosomal protein S15, cyclophilin type peptidyl-prolyl *cis-trans* isomerase and calcium-binding domains among these genes (S4 Table), suggesting again that the novel genes are associated with coding genes of potential importance in modulating molecular functions in the antennae.

We have summarized the variants/isoforms of the existing genes in S5 Table. Among our 390 variants, 42.82% (167) were isoforms of *G*. *m*. *morsitans* existing gene transcripts version 1.9 with stable community established structural and functional annotations and identity (ID) in VectorBase [28]. These variants are putatively associated with cellular or molecular functions that include protein transport, metal ion binding, neural signaling, oxidative regulation, development, cell degradation, gene expression regulation, response to environmental changes and olfactory roles such as odorant reception, gustatory responses and protein degradation. Putative functions of the rest of these variants (223) have not been annotated/assigned in the VectorBase [28].

### Novel gene differentially expressed in the antennae of male *G*. *m*. *morsitans* in response to attractant odor

Our assessment of differentially expressed transcripts revealed significant induction (3.5-fold change and FDR of <0.05) of GMOY014237.R1396 novel gene transcript in response to attractant exposure relative to the no-odor control (unfed). This (GMOY014237.R1396) is among the novel putative *G*. *m*. *morsitans* specific transcripts without any ortholog in any of the three (*M*. *domestica*, *D*. *melanogaster* or *An*. *gambiae*) genomes or homologues among Uniprot database genes [43] (S3 Table). Other attractant associated moderately induced (by at least 1.2-folds) novel transcripts included 1) GMOY014112.R1263 and GMOY014071.R1219, which were potentially associated with protein degradation and ribosomal RNA processing, and 2) GMOY014158.R1315 by the repellent associated putative organ development functions (S3 Table). However, these inductions were not statistically significant. Our GMOY014237.R1396 differentially expressed gene was functionally associated structural molecule activity as revealed by GO Term GO:0005198 associated with it in VectorBase [28]. We could not independently validate expression levels of the single differentially expressed genes using RT-qPCR [37, 50], due to limited biological resources/materials.

## Discussion

In this study, we annotated *G*. *m*. *morsitans* tsetse fly antennae-expressed genes in genomic loci supported by unmapped reads (without corresponding transcripts among existing gene models/annotation). These reads are typically discarded from further downstream analyses. We consequently identified 983 antennae-expressed genes in male *G*. *m*. *morsitans* as valid structural and putatively functional genes in the antennae. Among these, 592 gene models showed sufficient evidence for curation, validation and adoption for community use in VectorBase [28], and of these, 202 of the gene models were novel. We retained the rest of the models (391) in the database for subsequent curation due to lack of supportive evidence. Our current RNA-Seq libraries evidence had not been integrated into VectorBase database [28] at the point of annotation and thus some of the gene models exclusively supported by our libraries lacked sufficient in-house evidence for curation. Additionally, about 30% of our *de novo* assembled transcripts were not integrated in the predicted gene models/coding genes by MAKER computational pipeline [26], probably due to lack of supportive evidence from the Uniprot protein database [43], which we provided as protein evidence within the pipeline, due to our limited in-house computational capacity to process Metazoa subset of the non-redundant (nr) protein database in the National Center for Biotechnology Information (NCBI), previously used in the initial annotation of the *G*. *m*. *morsitans* genome using the same pipeline [13]. These *de novo* assembled transcripts might also have been components of noncoding RNA (ncRNA) genes in the genome. The Encyclopedia of DNA Elements (ENCODE) Program established significant transcription of DNA as RNA, vast majority of which are ncRNA [51]. We nevertheless improved the annotation of the *G*. *m*. *morsitans* genome by at least 4.55%, potentially adding to the 13,018 number of genes in the *G*. *m*. *morsitans* transcripts gene-set version 1.9 [28], and established that we can uncover new genomic/biological information from unmapped reads from the antennae-expressed *G*. *m*. *morsitans* transcripts. The significant (> 96%) mapping of the antennae expressed reads onto the genome indicate sufficient (quality) assembly of the reference genome. However, mapping statistics of the reads onto the transcripts points to 1) a major gap (incompleteness) in annotations of *G*. *m*. *morsitans* genome, 2) potential under representation of composition of genes present in *G*. *m*. *morsitans* genome, and 3) deficiency in annotation of some tissues specific/expressed genes in *G*. *m*. *morsitans*. Total number of annotated coding genes in *G*. *m*. *morsitans* is considerably smaller (by more than 50%) than for *D*. *melanogaster* [13], which may be due to genes that have not been identified and annotated in *G*. *m*. *morsitans* genome. Similar relationship has previously been observed between coding genes in avian and tetrapod genomes [52–54] and attributed to incomplete annotation of the avian reference genomes [55]. Our approach in annotation of the unmapped reads can be applied to improve annotations of draft genomes by annotation of tissue-specific genes that can in turn lend additional insight into molecular processes that underpin the physiological responses and related phenotype of the organism. While our findings revealed potential gap in *G*. *m*. *morsitans* genome annotation, the extent of this gap (genes misassembled or not annotated) and identification of the genes that miss in *G*. *m*. *morsitans* compared with *D*. *melanogaster* and related taxa/species remain to be established. However, this can be addressed by application of our approach in annotations of unmapped reads associated with tissue specific RNA-seq libraries as has recently been implemented in improving annotation of 9,206 genes and identification of 2,000 novel genes in the genome of *Rhodnius prolixus* Triatominae insect species [56].

At a functional level, this study provides us with new insights into the novel genes that potentially mediate molecular functions in the antennae of male *G*. *m*. *morsitans*. None of the orthologs or homologs of the novel genes in selected genomes (*M*. *domestica*, *D*. *melanogaster* or *An*. *gambiae*) and Uniprot database genes, respectively, were putative canonical chemosensory genes. The antennae are principal olfactory organs in insect with diverse sensilla [19, 57] expressing a myriad chemosensory genes. The absence of chemosensory genes among our novel genes could be tied to previous efforts that specifically focused on annotation of chemosensory genes [14–18], that potentially annotated most or all the existing chemosensory genes. Our finding therefore suggests likelihood of successful annotation of vast majority, if not all, of antennae associated chemosensory genes. Alternatively, our failure to annotate novel chemosensory genes could be due to limitations in our 1) annotations of some of the coding gene predicted by MAKER computational pipeline [26], and 2) *G*. *m*. *morsitans* tissues and physiological states (RNA-Seq library from only male of specific age from laboratory colony reared flies). Tsetse specific novel chemosensory genes might also be part of the 108 novel genes without orthologs in the selected genomes or homolog in the Uniprot database. This is supported in part by the apparent significant differential transcriptional responses in GMOY014237.R1396 novel gene in that category to attractant exposure relative to the no-odor control in the current study. However more insight into biological role of this gene (GMOY014237.R1396), can be better facilitated by validation of its expression using RT-qPCR [37, 50] and function using "empty neuron" heterologous expression system [33] that were not performed. Nevertheless, annotations of novel non-canonical chemosensory genes attest to enrichment of the antennae with genes that facilitate specific responses to other olfactory active compounds associated with active attraction to preferred hots, active avoidance of refractory animals, and other important roles of the antennae, including mechanoreception [21,23–25] and other regulatory processes that co-ordinate responses of tsetse fly to external stimuli. Thus, the novel genes identified included those associated with metabolic process, intracellular responses to extracellular signals, stress, regulation of cell cycle/growth, water homeostasis and diuresis and nervous system development that point to complex molecular processes in the male *G*. *m*. *morsitans* antennae. How these processes relate to phenotypic/behavioral and olfactory responses of the tsetse flies to odor is not clear but play a critical regulatory role in modulating such behavior. Further annotations of the missing genes in this tissue, including the novel putative *G*. *m*. *morsitans* specific genes, might provide further insight on the additional genes involved with mechanoreception and chemoreception typically associated with antennal functions in insects, including tsetse flies. However, such annotations would provide better resolution of the missing genes if RNA-Seq library originated from both gender and wild population of *G*. *m*. *morsitans* of various physiological states.

In conclusion, we have shown missing genes with biologically significant information can be annotated, from reads that do not align to existing gene transcript (unmapped reads), but specific to the *G*. *m*. *morsitans* genome. These genes can provide further insights on those that potentially mediate molecular functions in the antennae of male *G*. *m*. *morsitans*.

## Supporting information

S1 Table Blast hits of Trinity *de novo* assembled male *G*. *m*. *morsitans* unmapped reads transcripts/contigs against Uniprot protein database. (XLSX)

S2 Table Blast hits of Trinity *de novo* assembled male *G*. *m*. *morsitans* transcripts/contigs (from unmapped reads) against *G*. *m*. *morsitans* transcripts in VectorBase. (XLSX)

S3 Table Orthologs in fruit fly (*D*. *melamogaster*), housefly (*M*. *domestica*) and/or mosquito (*An*. *gambiae*) of novel transcripts expressed in male *G*. *m*. *morsitans* antennae. (XLSX)

S4 Table Blast hits of novel male *G*. *m*. *morsitans* antennae expressed genes against Uniprot protein database. (XLSX)

S5 Table InterProScan classification of protein domains of novel male *G*. *m*. *morsitans* antennae expressed genes. (XLSX)
